# Deciphering the molecular interplay and tumorigenesis in hepatocellular carcinoma through insights into FBXL6 and KRAS^G12D^

**DOI:** 10.1186/s40779-024-00515-w

**Published:** 2024-02-08

**Authors:** Qiang Cai, Quazi T. H. Shubhra

**Affiliations:** 1 Department of Neurosurgery, Renmin Hospital of Wuhan University, Wuhan, 430060 China; 2 Institute of Chemistry, University of Silesia in Katowice, 41-500 Chorzów, Poland

**Keywords:** Hepatocellular carcinoma, F-box and leucine-rich repeat 6 (FBXL6) activation, Kirsten rat sarcoma (KRAS)^G12D^ mutation, Mitogen-activated protein kinase (MEK)/mammalian target of rapamycin (mTOR) pathway, Metastasis

Liver cancer, particularly hepatocellular carcinoma, remains a formidable challenge in medical research and requires a deeper understanding of its molecular underpinnings. In a fascinating recent study published in *Military Medical Research*, Xiong et al. [1] revealed the complex roles of F-box and leucine-rich repeat 6 (FBXL6) and Kirsten rat sarcoma (KRAS)^G12D^ in the pathogenesis of liver cancer. This research offers critical insights into how these proteins contribute to hepatocellular carcinoma development and progression, potentially paving the way for targeted therapeutic strategies. This commentary analyzes the key findings from this study and their broader implications in oncology.

Cancer continues to pose a major challenge to global public health [2]. Liver cancer presents a multifaceted treatment obstacle with the sixth-highest prevalence and third-highest mortality rates worldwide [3]. It can be categorized into primary liver cancer, such as hepatocellular carcinoma, and secondary or metastatic cancers. This complexity is compounded by the liver’s tendency to develop metastases, which is frequently observed in various malignancies, especially in gastrointestinal cancers like colorectal adenocarcinomas. This trend is partly attributed to the unique dual blood supply of the liver, making it not only a common site for metastatic disease but also an ideal candidate for advanced interventional therapies. The inherently aggressive nature of hepatocellular carcinoma, combined with the crucial role played by the liver in metastasis, underscores the urgent need for innovative and holistic treatment approaches. These strategies must skillfully address both primary and metastatic liver cancers, reflecting the complex oncologic challenges of this organ.

The research conducted by Xiong et al. [1] represents a significant breakthrough in our comprehension of the molecular mechanisms of liver cancer, mainly focusing on the interaction between FBXL6 and KRAS^G12D^, as well as their roles in activating the mitogen-activated protein kinase (MEK)/extracellular signal-regulated kinase (ERK)/mammalian target of rapamycin (mTOR) signaling pathway. This pathway plays a crucial role in cell growth and survival, particularly in cancerous cells. Through an analysis of the interplay between these two proteins, this study sheds light on a pivotal aspect of liver cancer progression, providing a foundational insight that may pave the way for innovative therapeutic approaches.

Central to this study was the discovery of how FBXL6 activates both the wild-type KRAS and its mutant variant, KRAS^G12D^, through K63-linked polyubiquitination at the K128 site. This process, previously poorly understood, has now been revealed as a key mechanism for activating KRAS in liver cancer cells. It should be noted that aberrations in KRAS signaling are widely recognized as essential drivers of various cancers [4]. The significance of this finding lies in its potential to redefine approaches to targeting KRAS-driven cancers, especially hepatocellular carcinoma. Moreover, it provides valuable insights into hepatocellular carcinoma metastasis and progression by establishing a molecular foundation for understanding the aggressive nature of this cancer type.

The study further elucidated the role of FBXL6 in enhancing the aggressiveness of KRAS^G12D^-driven hepatocellular carcinoma and its propensity for lung metastasis. This insight is critical as it highlights the synergistic effect of elevated FBXL6 with KRAS^G12D^, leading to a more aggressive form of liver cancer. This synergy, specifically through the upregulation of proteins of relevant evolutionary and lymphoid interest domain 2 (PRELID2), introduces additional complexities to hepatocellular carcinoma development. It underscores the intricate molecular interactions involved in liver cancer progression and highlights PRELID2 as a potential therapeutic target. Notably, this study demonstrated the effectiveness of MEK and mTOR inhibitors in reducing hepatocellular carcinoma and lung metastasis, aligning with the growing body of evidence supporting their roles in treating various cancers [5]. This approach offers a promising strategy for hepatocellular carcinoma treatment, particularly for cases with specific molecular profiles, paving the way for more personalized and effective treatment regimens.

Lastly, this study revealed a critical correlation between high levels of PRELID2 and the activation of FBXL6/p-ERK/p-mTOR signaling pathway, which has been linked to poorer prognosis of hepatocellular carcinoma patients. This finding suggests that PRELID2 could serve as a biomarker for assessing the severity of hepatocellular carcinoma and offer a valuable tool for early detection and prognosis. The potential of targeting PRELID2 as a therapeutic intervention emphasizes the importance of this discovery, implying that interventions aimed at inhibiting this protein may yield more efficacious treatment strategies for hepatocellular carcinoma patients.

The study by Xiong et al. [1] represents a significant stride in liver cancer therapeutics. This finding highlights a critical shift towards a more personalized treatment approach that caters to the specific molecular features of hepatocellular carcinoma, aligning with the current trend of precision medicine to provide tailored and potentially more effective treatments for liver cancer patients. However, the study’s reliance on an orthotopic hepatocellular carcinoma tumor model in mice, while insightful, may not entirely replicate the intricate complexity of human hepatocellular carcinoma due to inherent biological differences. Future research would benefit from the inclusion of human-derived hepatocellular carcinoma models, such as patient-derived xenografts, to achieve a more accurate representation of the human condition. Furthermore, the study’s approach of using everolimus and trametinib as free drugs opens avenues for improvement through targeted drug delivery systems (DDSs). Targeted DDSs have the potential to significantly enhance drug potency, prolong plasma half-life, and optimize therapeutic indices which ultimately could lead to increased drug effectiveness, reduced side effects, and more precise tumor targeting [6]. The transition of these findings to clinical trials in human subjects is eagerly anticipated, as it will provide critical validation of these approaches in real-world medical settings.

In summary, this research marks a significant advancement in liver cancer research and offers valuable insights into the roles of FBXL6, KRAS^G12D^, and PRELID2 in hepatocellular carcinoma. It proposes innovative therapeutic strategies, establishes a new standard for hepatocellular carcinoma treatment, and lays the groundwork for future research in this field. This study is poised to revolutionize the management and prognosis of hepatocellular carcinoma, ushering in a new era for tackling this formidable disease.

## Data Availability

Not applicable.

## Abbreviations

DDSs: Drug delivery systems
ERK: Extracellular signal-regulated kinase
FBXL6: F-box and leucine-rich repeat 6
KRAS: Kirsten rat sarcoma
MEK: Mitogen-activated protein kinase
mTOR: Mammalian target of rapamycin
PRELID2: Proteins of relevant evolutionary and lymphoid interest domain 2
